# Perceptions of primary care in Korea: a comparison of patient and physician focus group discussions

**DOI:** 10.1186/s12875-014-0178-5

**Published:** 2014-10-31

**Authors:** Minsu Ock, Jung-Eun Kim, Min-Woo Jo, Hyeon-Jeong Lee, Hyun Joo Kim, Jin Yong Lee

**Affiliations:** Department of Preventive Medicine, University of Ulsan, Seoul, South Korea; The Center for Social Sciences, Seoul National University, Seoul, South Korea; Department of Public Health, The Graduate School of Konyang University, Daejeon, South Korea; Public Health Medical Service, Seoul National University Boramae Medical Center, 20 Boramae-ro 5-gil, Dongjak-gu Seoul, 156-707 South Korea

**Keywords:** Primary care, Focus group discussion, Quality of care

## Abstract

**Background:**

The primary care system in the Republic of Korea has weakened over the past decade and is now in poorer condition than the systems in other countries. However, little is known about how the two key players, patients and physicians, view the current status of primary care in Korea. This study aims to understand what problems they perceive in respect to the key components of primary care.

**Methods:**

We conducted two focus groups; one with six patients and the other with six physicians. We designed and modified the guidelines for each focus group discussion through repeated review and discussion among all authors and then we conducted the groups with a professional interviewer at Gallup Korea. After the focus groups we analyzed the verbatim transcriptions to identify specific meanings and potential implications.

**Results:**

From the study we identified that the patients and physicians did not have a correct understanding about the role of primary care. We also identified a significant discrepancy between their perception of primary care. In particular, the patient group perceived the quality of primary care to be poor and unsatisfactory while the physician group perceived the quality of primary care to be better in Korea than in other countries.

**Conclusions:**

The focus group discussions revealed that such discrepancies in perception have resulted from Korea’s distorted healthcare delivery system, undifferentiated roles among healthcare organizations, patients’ freedom of choice in selecting healthcare providers and other institutional factors. There are several steps that should be taken to promote primary care in Korea. First, we should undertake efforts to improve the quality of primary care provided by physicians. Second, we should inform the general public about using clinics instead of hospitals for the treatment of simple or minor diseases. Third, we should introduce a new compensation scheme to compensate physicians for services related to health education, disease prevention, behavioral change and nutrition consultation. Finally, we should provide additional reimbursement so that primary care physicians can extend their office hours to better meet the needs of patients.

## Background

Primary care plays an important role within healthcare delivery systems and influences the overall quality of care [1-3]. This is because primary care is the foundation of healthcare delivery systems and serves as the first contact point, or gatekeeper, for access to healthcare services. In addition, primary care plays a significant role in the coordination, comprehensiveness and continuity of healthcare services [1,4]. If primary care functions well within a system, it is estimated to meet 75 to 85% of healthcare needs, decrease unnecessary healthcare expenditures, and lead to positive health outcomes [5-8]. However, the primary care system in Korea has weakened over the past decade and is now in poorer condition than the systems in other countries [9-12].

System failures account for most of the issues in Korea. For example, the Korean government allows clinics to have inpatient facilities and also allows hospitals to provide a large scale of services to outpatients [13,14]. Patients in Korea can choose any clinic or hospital without a referral slip [15]. Therefore, clinics and hospitals are inclined to have as many patients as possible to maximize their profits and the relationship between clinics and hospitals has been characterized as competitive. In contrast, in Western countries, clinics generally care for outpatients and hospitals mainly care for inpatients. If a patient wants to see a doctor in a hospital, he or she first consults a primary physician and receives a referral letter before going to the hospital. The patient is then discharged back to the primary physician after the conclusion of hospital treatment. Thus, the relationship between clinics and hospitals can be viewed as collaborative.

Delivery providers’ overlapping roles and patients’ freedom of choice in terms of choosing care providers can weaken the function of primary care in an undifferentiated healthcare delivery system. To understand this problem in Korea, it is important to investigate patients’ and physicians’ view of primary care, their perception of strengths and weaknesses in respect to the key components of primary care and any potential barriers they experience that inhibit the function of primary care [16,17]. Thus, this study aims to understand the current status of primary care in Korea by comparing the perspectives between receivers (patients) and primary care providers (physicians). A qualitative study using focus group discussions (FGDs) is an appropriate mechanism to understand the differences and similarities that exist between patients and physicians in terms of the key components of primary care.

## Methods

### Study design

FGDs have been used in health research to explore the in-depth perspectives of study participants. An FGD is defined as “a carefully planned discussion designed to obtain perceptions on a defined area of interest in a permissive, nonthreatening environment” [18]. In this study, the FGDs were used to facilitate participants’ expression of their perceptions about primary care. We conducted two focus groups; one with six patients and the other with six physicians. The guidelines for the patient and physician FGDs were designed and modified through repeated reviews and discussion among the authors. Because this study aimed to identify the strengths and weaknesses of the Korean primary care system based on the experience of key stakeholders, our guidelines included semi-structured questions based on the five key components of primary care – first contact, accessibility, coordination, comprehensiveness and continuity.

At first the questions were roughly listed based on our literature review and current primary care issues in Korea, and then the questions were categorized by the specific topics that represent the key components of primary care. We created the questions to ascertain a diversity of perceptions about primary care and to make consistent and reliable comparisons between the patients and physicians (Table 1). Only the authors knew the guidelines for the purpose of study, and the moderator used the question list during the interview regardless of the guideline categories. The authors also met several times to finalize the desired number of participants, characteristics of participants, and the method for recruiting participants.

**Table 1:** **Guidelines for FGD**

	**Physicians**	**Patients**
Icebreaking	• Motivation and satisfaction about opening clinics	• Personal health condition and usage of clinics
The key components of primary care	**First contact**	
	• Do you think patients are more likely to contact secondary and tertiary hospitals than primary care?	• Do you have a favorite clinic to go to when you are sick or need medical consultation? If not, how do you feel?
	• Do you think your clinic is ready to provide sufficient primary care?	• If you just found out that one of your family members has chronic disease, where would you take her/him to?
	**Accessibility**	
	• How do you operate your medical consultation other than daily schedules? (e.g., consultation at nights, weekends, or holidays). If not, why?	• Do you think clinics in your neighborhood provide sufficient daily hours for medical services?
		• Have you ever made a reservation for medical services at the clinics in your neighborhood?
		• Have you ever had a medical consultation via phone call?
		• Do you think your payment for the medical services in your neighborhood clinics is appropriate?
		• Have you ever been denied by your neighborhood clinics?
	**Coordination**	
	• Have you ever referred your patients to secondary or tertiary hospitals? When? Why or why not? How?	• When you have health problems, do you have any issues when you decide the types of clinics you should go to? And departments of the clinics you should go to?
	• Have you ever referred your patients to community healthcare centers, other healthcare providers, or other community-based organizations except the clinics?	• Have you ever asked for medical treatment requests to your neighborhood clinics?
	• How well do you coordinate medical services for your patients?	• Have you ever been referred to other clinics or community health service providers/centers?
	• What resources and actions do you need for improving the coordinating role of primary care?	
	**Comprehensiveness**	
	• What are your ranges of treatments, diagnoses, and operations you provide to your patients?	• Have you ever received medical examination or cancer screening at your neighborhood clinics?
	• How do you educate your patients in terms of their healthy life style such as: Do you know the range of medical services that the neighborhood clinic can provide you with? For example, ranges of treatments, diagnoses, and operation	• Have you ever participated in education for health behaviors at your neighborhood clinics? What are your thoughts on such education?
	**Continuity**	
	• How do you manage your patients with chronic diseases for regular checkup and treatments?	• When you have similar symptoms, do you visit the clinic that you usually use, or do you visit several clinics?
	• Have you ever asked for medical records from your patients for appropriate medical treatments to understand their medical history and information for treatments?	• How do your neighborhood clinics know your medical history and medical information?
Primary care issues in current policy	• What do you think about	• What do you think about
	○ Double count system for working on a closed Saturday	○ Health cooperative
	○ Chronic disease management system	○ Family doctor registration program
	○ Jointly run clinics	○ Chronic disease management system
	○ Other strategies to improve primary care	○ Other strategies to improve the primary care

### Participants

Six patients were purposively selected for the FGD (Table 2). Because of limitations in accessing a patient population with the desired primary care experience, the doctors in our research team randomly asked the willingness of their patients to participate in the study. In recruiting the potential participants, we considered their demographic characteristics to ensure a diversity of gender, age and prior experience with the primary care system. These selection criteria were established to avoid any previous bias from the participants. The patients who were willing to participate in the study were sufficiently informed about the study’s purpose, that no harm would be caused by study participation, and that their privacy would be protected. The average patient age was 44.7 and ranged from 22 to 66. All three women were housewives with children. Apart from one retired man, the other two men had jobs. Most participants had a higher level of education, were married, and had no chronic diseases. One patient reported having a prior diagnosis of kidney cancer.

**Table 2:** **Characteristics of patients**

**Number**	**Education**	**Child**	**Chronic diseases**
1	Community college	2 (Toddler & Preschool student)	None
2	University	2 (Toddler & Higher school student)	None
3	Community college	2 (High school student)	None
4	-	0	None
5	University	2 (College students)	None
6	-	-	Kidney cancer

Similarly, the physician FGD included six physicians who were purposively selected from the Korean Physicians’ Association directory (Table 3). As opposed to the patient selection criteria, for this FGD we considered factors such as the physicians’ clinic location, size, capacity, and years of operation. Over one week, we contacted the physicians drawn from the list, based on the selection criteria, and finalized the group based on the physicians’ willingness to join the FGD. Three physicians have operated their clinics for less than ten years while the other three have been running their clinics for more than ten years. All six physicians majored in internal medicine. Only one of the physicians was female and only one physician worked at a jointly owned clinic. Except for two physicians who came from the same community, the other physicians were from different communities. A written informed consent for both FGDs participation was obtained from each participant before the FGD and an incentive (around 90 United States dollars) was given to each participant after the FGD.

**Table 3:** **Characteristics of physicians**

**Number**	**Years since opening clinic**	**Number of staff**	**Average number of patients per day**	**The ratio of patients with chronic diseases**	**Medical service provision on nights and holidays**
1	Over 10	3	70	50%	No
2	Over 10	2	70	35%	No
3	Under 10	10 (Joint opening)	120	20%	No
4	Under 10	2	70	60%	No
5	Over 10	2	65	50%	No
6	Under 10	3	70	90%	No

### Procedure

The patient and physician FGDs were both conducted for three hours in an observation room equipped with a one-way mirror and audio and video recording at Gallup Korea. There were several FGD operators, including one professional interviewer from Gallup Korea who served as an independent moderator of the focus groups; one interview assistant from Gallup Korea who was responsible for the audio recordings, video recordings and keeping written records of any significant nonverbal behavior; and four research team members who participated as observers. Except for the interviewer, the interview assistant and the other research team members stayed behind the one-way mirror. Focusing on the discussion topic, the moderator led participants’ natural discussion according to the research guidelines.

While the group discussions were conducted, the moderator confirmed if the participants had any issues and asked clarifying questions, especially of the quiet participants. In the case of the six patients, the moderator explained the meaning of primary care and differentiated it from secondary and tertiary care. Additionally, because the moderator had experience with group discussions, he naturally introduced the aim of study, tried to equally assign the speaking order, encouraged interaction among the participants, and stressed that every participant’s opinion was valuable. Five minutes before the end of the discussion, the moderator entered the room behind the one-way mirror to meet with the research team and check if there were any additional issues to confirm or clarify before the interview ended. Each FGD was audio-recorded.

### Data analysis

The audio-recordings from the FGDs were transcribed verbatim into an electronic transcript (Microsoft Word) in Korean language. After analysis, the Korean language transcripts were translated into English language for presentation in this paper. We conducted a content analysis by analyzing the verbatim transcripts and identifying specific meanings and potential implications, in accordance with the guidelines. Content analysis is defined as “the systematic, objective, quantitative analysis of message characteristics” [19]. To identify specific themes from the content that the participants repeatedly stated, two authors read the transcriptions repeatedly, each time writing memos and highlighting sentences with significant quotes. Each FGD transcript was respectively coded using directed content analysis [20].

The codes were categorized according to the key components of primary care, based on the similarity of the codes. These codes were then merged as themes according to the predetermined coding scheme written in the guidelines. To improve reliability, two coders undertook a reiterative content analysis process that involved adding and merging key themes from the coded content. In addition, the research team also reviewed the coded themes and held discussions with the coders to reach conclusions regarding the key study findings. Computer-assisted qualitative data analysis software, NVivo 10, was also used for the analysis. The major themes from each FGDs were then compared to identify the similarities and differences between the physicians’ and patients’ perceptions of primary care.

### Ethical approval

This study was approved by the Institutional Review Board of Asan Medical Center (S2013-1338-0002).

## Results

The data analysis resulted in 217 codes from the patient FGD and 250 codes from the physician FGD. The codes were then sorted into 6 categories that included the five key components of primary care and a new emerging component, quality of care. Thus, the final extracted set of common components of primary care in this study included quality of care, first contact, accessibility, coordination, comprehensiveness, and continuity. Based on these six categories, we found specific subthemes under the key primary care components and then compared the similarities and differences existing between the patients’ and physicians’ perceptions of the current status of primary care (Table 4).

**Table 4:** **Main content comparison by themes**

**Key component of primary care**	**Themes**	
	**Patients**	**Physicians**
Quality of care*	• Distrust in primary care physicians(-)	• Difficulties in meeting diverse needs of patients(-)
	• Trust in the hospital care system(+)	• Better than other countries(+)
First contact	• Trust-based relationship with primary care physicians(+)	• Unawareness of patients(-)
	• One-stop service provision(-)	
Accessibility	• Extending doctor’s office hours(-)	• Needlessness of extending doctor’s office hours(-)
	• Absence of duty clinic system(-)	• Insufficient financial compensation(-)
Coordination	• Unawareness of patients(-)	• Competition to acquire their own patients(-)
		• Insufficient financial compensation(-)
		• Difficulties in meeting diverse needs of patients(-)
		• Limited coordinating role(-)
Comprehensiveness	• Unawareness of patients(-)	• Insufficient financial compensation(-)
Continuity	• Follow-up management(+)	• Not my duty(-)
		• Insufficient financial compensation(-)

*Newly added key components of primary care.

(-) represents a weakness of the key primary care function listed on the left column.

(+) represents a strength of the key primary care function listed on the left column.

### Quality of care

Patient participants perceived that the current level of primary care in Korea is relatively poor, especially in terms of the quality of physicians. For example, patients generally considered that doctors in clinics are less advanced in their understanding of medical services than those in hospitals. These perceptions revealed patients’ doubt and distrust regarding the quality of primary care.

- *Distrust in primary care physicians*

*Moderator: Are clinics less reliable than national university hospitals?*

*Patient 6: Yes, because, I think the doctors opened their clinics when they fell behind from the hospital context; they could not survive in the mainstream of the big hospital.*

*Ellipsis*

*Patient 2: In my recent life, my child got a cold, and I took her to the clinic several times. However, she did not get better. I think, the old doctor, who is a woman, treated only on the basis of her own experience, without the newest information.*

*Patient 5: Did she always get the same treatment?*

*Patient 2: Yes, continuously repeated…*

*Patient 5: Always the same prescriptions whenever I visited*

*Patient 2: I think the doctors in clinics may have less opportunity to obtain recent medical information on a regular basis.*

*Patient 5: It is seen as inaction.*

Alternatively, the participating patients trusted the medical services from secondary and tertiary hospitals, which have more collaboration across diverse professional departments. In other words, the patients perceived that physicians working at secondary or tertiary hospitals are more outstanding than those in clinics and that the hospital medical system is more advanced than the primary care clinic system.

- *Trust in the hospital care system*

*Patient 2: I think, rather than doctors’ individual skills and professions, the quality of care depends on the hospital system, which has more capital to invest in better facilities and devices. Because of the better environment for collaboration among diverse professions, the personal skills of the doctors can gradually advance.*

*Ellipsis*

*Patient 2: One more thing I want to say, communication among doctors is a matter of the hospital system. The amount of time a patient meets doctors in hospitals, such as university hospitals, may be less than the meetings in clinics, but many doctors in the hospitals may try to examine one patient’s case through diverse perspectives within the systematic hospital environment. Such benefits from hospitals are more reliable.*

However, the participating physicians explained that the prejudice of patients is because of inappropriate broadcasting by the media and problems within the medical service delivery systems that have made the patients perceive that the quality of care in hospitals is better. The physician group believed that the quality of primary care in Korea is not lower than in Western countries.

- *Difficulties in meeting diverse needs of patients*

*Physician 3: Even though there are many primary care clinics, all of those clinics cannot satisfy the patients’ expectations. I understand why the patient perceives clinics with less reliability.*

- *Better than other countries*

*Physician 5: In other countries, the primary care quality cannot be better than the hospitals. Anywhere you go! But the only reason that primary care should be the first line in the delivery system is because of the effectiveness.*

*Ellipsis*

*Physician 2: I do not think the quality of primary care in Korea is poorer than in other countries. Actually, the primary care clinics in foreign countries only have small desks! There is no primary care clinic as good as in Korea.*

*Ellipsis*

*Physician 5: Originally the quality of primary care in Korea was better than in America. Just ask Korean Americans!*

### First contact

Most patients in Korea are more likely to go to hospitals directly without screening by primary care physicians. Some patients who participated in the FGD also went to hospitals directly without consultation in a clinic, but others used clinics as their first contact point depending on their relationship with the primary care physician.

- *Trust-based relationship with the primary care physician*

*Moderator: Do you use the clinics in your community?*

*Patient 3: Yes, I can easily access the community clinics. Because we have no big diseases, my child just has simple colds ….And we could not go to the secondary or tertiary hospitals without passing the primary care. So the primary care clinic is first…*

*Ellipsis*

*Moderator: How did the clinic physician become your family doctor?*

*Patient 3: When my child was young and sick, I frequently visited the clinic located near my town. So we could have a longer consultation time.*

*Moderator: Because your physician knew your child?*

*Patient 3: Yes.*

*Moderator: Why was your first visit there? Just because it’s nearby?*

*Patient 3: Yes, very close*

*Moderator: If so, why did you keep going only to the clinic?*

*Patient 3: The physician was very nice and consulted very well. Like he told me what he can do and what he cannot do and recommended me to go to see other specialists, such as a dermatologist and ENT doctor.*

*Moderator: Exactly.*

*Patient 3: Yes, he sent me to the right places.*

*Moderator: He sent you to other clinics or hospitals if necessary and he provided you with good consultation…That was because you used the clinic as your family doctor?*

*Patient 3: Because, you know, the symptoms of illness were always different every day. But, still he explained step by step why the different symptoms or illnesses were happening, because he knew my child for a long time, he could guess whether the illness would disappear soon or if my child would need to endure a little more… and gave the prescription with low dosage….*

*Moderator: He knew very well about your family.*

*Patient 3: Yes, very precisely.*

*Moderator: How long have you been going to the clinic?*

*Patient 3: Ten years.*

*Moderator: Because of your child only?*

*Patient 3: Yes, from when my child was very young…the clinic has the consultation history of my child.*

*Moderator: The clinic has everything?*

*Patient 3: Yes, like x-ray…. He determined whether the cough is from cold or bronchitis because he knew my child for a long time and remembered my child’s physical composition.*

However, most of patients usually visited the secondary or tertiary hospitals to receive consultation about all of their diseases at once, rather than going through primary care. Even though the patients used the clinics sometimes, they distrusted the clinics because the clinics have poor medical equipment.

- *One-stop service provision*

*Moderator: What about you?*

*Patient 6: I definitely go to the big hospital.*

*Moderator: Seoul National University Hospital?*

*Patient 6: Yes, I had surgery within 3 days after diagnosis. I went to the clinic in my town first, but the clinic could not find any disease. But I asked the clinic doctor to examine further and finally he found I had a disease…so I went directly to Seoul National University Hospital.*

*Ellipsis*

*Moderator: Do you have any issue when you go to the clinic?*

*Patient 1: There are some clinics that are well equipped, but some clinics are not, such as x-ray…The pediatrician I usually go to have no x-ray machine. If my daughters have a cough, the physician can diagnose only by using a stethoscope. So I am little doubtful that it’s okay.*

*Ellipsis*

*Moderator: If you had high blood pressure of 150 mmHg, can you get medicine from the clinic monthly and take the medicine regularly?*

*Patient 1: The first time I got a diagnosis, I had to go to the big hospital where I could get multiple examinations to assess for other veiled diseases. Who knows, I could have had another disease, so I am sure I had to go to the hospital for the first examination*.

*Ellipsis*

*Patient 3: However, I think there is something lacking, because there is some inconvenience not to be examined at the same time.*

*Moderator: Can you tell us in more detail?*

*Patient 3: If I go to the hospital, like St. Mary, when I get an eye examination, I can get a blood test while I am waiting for the result of the examination. After the blood test the hospital can directly refer me to other hospital departments, like endocrinology or neurosurgery, if my examination identified some problems. However, in clinics, I have to be in and out several times to diagnose my diseases.*

The physicians also recognized that patients preferred hospitals over clinics as the first contact. In addition, the physician group described that the patients’ preference resulted from their wrong perceptions about the clinics as well as structural problems in the healthcare service delivery system, which allows patients to easily access secondary and tertiary hospitals.

- *Unawareness of patients*

*Physician 2: Actually, some patients can choose whether they go to clinics for their mild disease or go to the hospitals for their severe illness. However, I think, most patients have no ability to determine whether their illness is mild or severe. Just think about your shopping. I am sure that you think the expensive thing is better when you have no idea about that particular thing. That is the similar perception when the patients go to hospitals. Because they do not know well about their diseases, they want to go to hospitals that have many doctors. The system, which can educate patients to change such behaviors, is necessary.*

*Ellipsis*

*Physician 1: Should change perspectives of people in general. Primary care is sufficient for a simple illness. However the problem is that regardless of the patient’s illness, they may choose to go to hospitals.*

### Accessibility

The participating patients recognized that they could easily access primary care in terms of proximity and cost. However, they were not satisfied with the accessibility of primary care services during weeknights, weekends, and holidays.

- *Extending doctor’s office hours*

*Moderator: What do you think about the doctor’s office hours in your clinics?*

*Patient 5: Not satisfied.*

*Moderator: With what?*

*Patient 5: Middle or high school students like my child should get to school by 8:30 am, but the clinics usually open at 9 am or 9:30 am. So my child had to skip the first class to go to the clinic. I think the clinics should open at least around 8:30 am.*

*Patient 1: Not only the child, but also office workers like my husband could not go to the clinic because the clinic was already closed soon after he left work. Actually, the child is usually sick at nighttime, so I think the clinics should stay open until 8 pm. If the clinic is open late, I do not have to hurry to the emergency room at night.*

*Ellipsis*

- *Absence of on-duty clinic system*

*Moderator: What about opening on weekends or holidays?*

*Patient 6: I wanted it before, but now I am okay if they are closed.*

*Moderator: Why are you okay?*

*Patient 6: Because they also need to take a rest…*

*Moderator: The physicians should take a rest?*

*Patient 2: It would be good if the clinics were open on weekends, but I do not think they can open every day. It is necessary to have a social system in which the clinics share the duty to work on weekends and holidays, because patients always exist even on holidays.*

The physician group had different thoughts about extending clinic office hours. The physicians generally expressed negativity about providing medical services during nights and holidays. They were also against an on-duty clinic system because they perceived that patients can sufficiently use existing providers or access the emergency medical service. Without increasing compensation, the physicians conveyed that extending doctors’ office hours was not realistic.

- *Needlessness of extending doctor’s office hours*

*Physician 1: I think there is a discrepancy between patients’ and doctors’ perspectives. From the physician perspective, why is expanding doctor’s office hours necessary? Because patients can go the 24-hour emergency center of the university hospital! And why are on-duty clinics necessary? Patients can easily go to the hospital by taking a taxi! Furthermore, patients can get prescription medicine after medical treatment there! There is no country in the world like Korea where patients can go to the general hospital so easily! Again, why are on-duty clinics needed?*

*Ellipsis*

- *Insufficient financial compensation*

*Physician 2: From the patient perspective, opening the clinics 24 hours would be good for the patients. But that is only true for the patients! As for us, we cannot open 24 hours, 365 days for those patients. If the patients are not sick, they might not come. As already mentioned before, a system to support the on-duty doctors would be better. If not, the treatment at nights from 6 to 9 pm in general would be inefficient.*

*Ellipsis*

*Moderator: What if we have no system to support the on-duty doctors?*

*Physician 1: It cannot be operated. It will run deficit.*

### Coordination

The function of coordination in primary care is that physicians refer their patients to other medical institutions after appropriate medical treatment. However, the patients in this study did not recognize the coordination function of primary care, and they only understood primary care as where to get a referral slip to go to secondary or tertiary hospitals. Moreover, most of the participating patients preferred choosing clinics or hospitals by themselves, but they also reported that they had difficulties in choosing them.

- *Unawareness of patients*

*Moderator: How do you determine which department of the hospital you should go to when you are sick?*

*Patient 2: I asked my wife.*

*Moderator: What about Patient 1?*

*Patient 1: If I had a problem with my stomach, I went to see the internal medicine doctor. If I coughed, I went to see the ENT doctor. So depending on where I had a problem. It was simple.*

*Ellipsis*

*Moderator: Have you ever heard about a referral slip to go to the hospital?*

*Everybody: Yes.*

*Moderator: Have you ever requested a referral slip from the clinic?*

*Patient 5: Yes.*

*Moderator: When?*

*Patient 5: I never requested it, but the physician gave one to me when I had a skin allergy. However, the referred clinic was dermatology and it looked unreliable.*

The physician group expressed their difficulties in coordinating medical services. In respect to patient acquisition, some physicians preferred not to send their patients to other clinics. Moreover, the physician group noted that Korea’s current coordinating system allows patients to go to hospitals without a referral slip from a primary care physician.

- *Competition to acquire their own patients*

*Physician 5: When clinics first open, they are open 365 days and even at night to have more patients. That is a bloody competition. If not, there is no way for them to survive. So, in such a case, physicians must not let go of their patients to other clinics.*

- *Insufficient financial compensation*

*Physician 1: We do our best to consult the patients, but the compensation system is limited. For example, we are expected to provide $5 in services to patients even though they paid $2-3.*

*Physician 2: We cannot operate well as the gatekeeper because patients have the freedom of choosing secondary or tertiary hospitals. In fact, the tertiary hospital should be accessed through the family medicine doctor’s referral, but patients can directly go to the secondary hospital.*

*Moderator: If they want…*

*Physician 2: Like in Australia or other Western countries, the national healthcare delivery system should be changed to promote the function of coordination.*

*Moderator: Unless the system is created….*

*Physician 2: Cannot expect the coordination of primary care.*

*Moderator: You mean, unless the institutional issue is solved first, we cannot overcome the weak coordinating function of primary care?*

*Physician 5: Yes, there is no efficient way to coordinate healthcare services with national medical resources. Only coordination across individual primary care physicians exists.*

When the physicians referred their patients to other clinics, they had difficulty perceiving the patients’ opinions and were worried about losing trust-based relationships with their patients due to the referrals. One of participating physicians described his adaptive behavior in which he was more likely to refer his patients when a good outcome was expected, rather than when the referral was medically necessary.

- *Difficulties in meeting diverse needs of patients*

*Physician 2: I introduced one of my relatives to the S hospital. At that time, I asked and asked for a favor from the hospital, despite the hospital’s busy schedule, they finally found a time for him to be examined, but....... he did not visit the hospital! I have experienced such cases many times, so it was a burden for me to refer him. Not all referrals are good cases.*

*Ellipsis*

*Physician 3: That is right! It is covered when I referred my patients to another physician who acquainted each other. I usually asked my patients if they preferred clinics or hospitals, and then I referred them to where they wanted to go. Also, I usually see cancer patients, so in case of good prognosis, I refer them; otherwise, if there is a poor prognosis, I do not refer them.*

The physician group also expressed that they have no interest in using social resources as a component of primary care. Some physicians referred their patients to public healthcare centers, which provide free public health education programs such as anti-smoking education. In the case of patients with tuberculosis, because the disease requires much administrative work and a lower consultation fee, the physicians referred them to public health care centers. However, most physicians believed that public health care centers and other social service organizations provide lower quality services than the primary care clinics.

- *Limited coordinating role*

*Moderator: Have you ever referred your patients to other community service centers, such as public health care centers and social service centers other than secondary or tertiary hospitals?*

*Physician 2: Yes, to the public health care center.*

*Moderator: For what did you refer them to the center?*

*Physician 2: I knew the public health care center provides free anti-smoking education with free anti-smoking patches. Just for stopping smoking. That is it.*

*Ellipsis*

*Physician 2: We do not refer our patients to the public health care centers for medical treatment. The individual primary care physician is more professional than the public healthcare service providers.*

*Moderator: What else? Do you have other experiences referring or introducing social resources to your patients?*

*Physician 1: I referred a patient with tuberculosis to the public health care center. Such patient cases require me to do too much administrative work, such as regular electronic record updates. So, I wanted to send that patient to the public health care center.*

### Comprehensiveness

The patient group generally had a positive perspective that clinics provide patients with health consultations and education programs beyond medical treatments. However, they were not sure whether the clinics had to provide comprehensive educational programs to the community.

- *Unawareness of patients*

*Moderator: So, the program organizer does not matter; such as a church or public office?*

*Patient 2: Right, it does not matter which organization provides the programs.*

*Moderator: Does it matter who the educator is?*

*Patient 5: Who the instructor is, is more important.*

*Moderator: A real professional instructor is more important?*

*Patient 6: Yes, an expert should come to educate.*

*Ellipsis*

*Patient 2: A community church, not a clinic, provided health education programs and invited doctors. The next subject of the education program is for expecting mothers who will deliver soon. There is a lot of interest in education for mothers and there is a high participation rate. I think if the neighborhood clinics provided such programs, they would gather high attention like the church’s programs. However, I think the clinics do not have enough capacity to provide such classes to educate a big audience.*

However, the physician group perceived that they only provided health consultation and education for the purpose of advertising their clinics. Because there was no category to pay for patient consultation and education, the physicians described that they had no incentive to provide education and consultation for community patients. Nevertheless, the physician group agreed that it was necessary to provide programs for disease prevention and health management as part of the systemic approach to service provision.

- *Insufficient financial compensation*

*Physician 1: This issue is also related to the issue of medical fee compensation. The only item we receive compensation for is to manage chronic diseases. Special care is required for patients with chronic high blood pressure and diabetes, but there is no consultation fee. There is no consultation fee if the patients do not get direct prescriptions, despite spending many consultation hours. I have no idea what I have to do when my patient comes to see me for a health consultation and a prescription is not required. If there is no illness, and no prescription, I cannot charge them. The patient also wanted to pay for a thirty minute consultation, but there is no category I can use to charge them for such a health consultation. No diseases, no consultation fee!*

### Continuity

Continuity means that a patient has a sustaining relationship with his or her primary care physician. To continue the relationship, most of the patients expressed their positive thoughts regarding notification services to remind them about their next clinic visit by texting, calling, and emailing.

- *Follow-up management*

*Patient 1: In the case of medical examination or vaccination for infants, which are necessary weekly, monthly, or bimonthly, mothers often forget. So, I would like to receive text messages from the clinic to remind me about the exam schedule.*

*Patient 5: In my case, I had a thyroid surgery scheduled at a clinic, so I got a text message about the next check-up date. Also, I got a message from my maternity hospital to remind me to have a uterine and breast cancer examination.*

*Moderator: So, do you like to receive those services?*

*Patient 5: Absolutely, it is appreciated.*

On the other hand, the physician group generally did not perceive the necessity of notification services to remind patients of their next visit. They believed that the patients themselves were more responsible for remembering their regular clinic visits.

- *Not my duty*

*Physician 2: If the patients are given a specific schedule for administering medication, such as patients with high blood pressure, they should revisit the clinic when they need a prescription refill. In such a case, it would be redundant for me to send them text message reminders about their next visit.*

*Moderation: It is a redundant service?*

*Physician 2: Due to new privacy regulations, without consent agreement, we cannot text them!*

*Moderator: Is that a complicated procedure?*

*Physician 2: As I already mentioned before, it depends on the willingness of the patient! If the patient wanted to quit visiting the clinic at the end of this month, receiving text messages from the clinic can annoy the patient. If patients have a real willingness to be treated, they would come anyway!*

- *Insufficient financial compensation*

*Moderator: I heard that one of the patients was happy when they received a reminder message from the big hospital about their next visit. How would they feel if they receive a message from the clinic?*

*Physician 5: The big hospital has a lot of staff and money, so the electronic system or administrative department can manage customer service. However, the clinics have few staff. A notification service regarding the next visit can be another burden.*

*Moderator: So, do you have any good examples about providing such services to your patients?*

*Physician 2: Some patients do care. However, I think the big hospitals have sufficient capacity to respond to patients’ complaints and any other customer issues, but in my clinic, it’s just me! I have to do everything alone. It is very stressful.*

## Discussion

Korea’s current primary care system is struggling because of the overlapping functions between hospitals and clinics and from patients’ freedom to choose primary care providers [10-15]. These issues can weaken the function of primary care and lead to an undifferentiated healthcare delivery system between primary care providers and hospitals. Therefore, we wanted to investigate how the two key players, patients and physicians, evaluate the current status of primary care. If the two players really consider Korean primary care to be inadequate, we wanted to identify the specific advantages or problems for fulfilling the key functions of primary care. From the two FGDs, we added a new component of primary care, the quality of care, to the five key components that we began with first contact, accessibility, coordination, comprehensiveness, and continuity. We also found sub-themes within each key component of primary care which revealed the strengths and weaknesses of various primary care functions.

From the study, we were able to confirm that a significant discrepancy of perception regarding primary care exists between physicians and patients. Moreover, the FGDs showed that such discrepancy of perception was contributed by the distorted healthcare delivery system, undifferentiated role among healthcare organizations, patients’ freedom of choice of healthcare providers and other institutional factors. First of all, the patient group perceived the quality of primary care to be poor and unsatisfactory while the physician group perceived the quality of primary care in Korea to be better than other countries. The patient group had doubts about the quality of primary care physicians and found the primary care provided by hospitals to be more reliable. Conversely, physicians believed that patients’ expectation that the quality of primary care to be on par with hospitals was excessive and said it was difficult to meet the diverse needs of all patients.

This discrepancy in perception exists because patients in Korea can use outpatient hospital services for frequently encountered, simple and minor diseases without having to obtain referrals from primary care physicians [14,15]. In other words, patients can freely choose between clinics and hospitals with only minimal difference in cost for treatment of the same condition. Hence, from the patients’ perspective, they can expect about the same quality care from both clinics and hospitals. Especially for simple or minor diseases, the medical care provided by the clinics should not be much different from what the hospital provides. Yet, the patients’ subjective views and satisfaction level are higher for the hospitals because they believe that good facilities and equipment imply a better quality doctor [21].

Conversely, the physicians believed that the quality of medical care they provided is better than the care provided in other countries. This view was based on the fact that most primary care physicians in Korea are specialists rather than general physicians or family medicine doctors [4,14]. The physician FGD group considered the service provided by the primary care providers – the specialists – to be more diverse than in other countries and they also perceived the facilities and equipment in clinics to also be better. Therefore, the patient group evaluated the quality of medical care offered by the clinics based on their comparison of services provided in clinics and hospitals, rather than based on the qualification of primary care physicians. If the patients found the quality of care to be different between the two, they thought the quality of primary care was lower than hospitals. The physicians, on the other hand, determined the quality of primary care to be high if the doctors were more educated and the clinics had better facilities and equipment compared to other countries, as opposed to emphasizing the role of primary care in coordinating medical service and providing continuous of comprehensive services for patients.

Our findings suggest that the patients and physicians in Korea evaluate the quality and core attributes of primary care without fully understanding the true purpose of primary care. This inclination was also noticeably present when we asked the two groups to evaluate a key component of primary care – the first contact point. Some of the patients indicated a positive view towards the role of primary care as the first contact point, but other patients did not agree with having to go through a primary care clinic when one-stop services are available at hospitals. This can be interpreted as the patients believing that it is rational for someone with a non-chronic illness to use primary care service based on trust, but for someone with a more serious illness to seek hospital outpatient services because the effectiveness is perceived to be higher. While one of the core functions of primary care is to serve as gate-keeper to medical care, our findings show that this function is failing, similar to what research in other country’s has found [22,23]. In this regard, the physicians admitted that primary care has responsibilities beyond serving as first contact point, but they blamed their poor gate-keeping results on the ignorance or misunderstanding of patients, the referral system which allows patients to directly access the hospital without first going to a clinic and failures of the healthcare delivery system. These physicians acknowledged that there are substantial systematic issues, but they should also reflect on whether physicians have been serving the best interests of patients.

When the patients were asked about primary care accessibility, they indicated that physical and economic barriers were less of a problem than office hours. Patients would prefer to have extended office hours during nights, weekends and holidays. However, the physicians were very hesitant about offering services during non-traditional business hours primarily because there are little monetary incentives for offering additional office hours. Additionally, quite a few clinics do offer extended office hours even though the number of these clinics may not be significant. Under the current payment scheme, clinics should not be forced to work during nights, weekends and holidays, although such argument may cause debate. Improving physician reimbursement or other incentives is one way to meet the patients’ demands for better accessibility while also catering to the physicians’ concerns.

Of the six key components of primary care, the patients and the physicians expressed clear understanding about quality of care, first contact, and accessibility. However, for the remaining components – coordination, comprehensiveness, and continuity – the patients supported having these functions of primary care strengthened, but did not necessarily believe that these functions were an essential part of primary care. Only a few patients correctly understood that the roles of primary care physicians include having good knowledge of patients’ health conditions, making referrals that direct patients to appropriate health care services, receiving re-referrals, continuous management of chronic diseases, and providing comprehensive services that include health education and consultation. Some patients even mentioned that their reason for choosing hospitals over clinics was to receive one-stop hospital services. This implies that patients consider hospitals to harness coordination, comprehensiveness and continuity. In contrast, the physicians had better understanding of the functions of primary care - such as coordination, comprehensiveness and continuity - but they were not accustomed to, and felt uncomfortable offering, these services.

Although physicians are aware of these functions, they are not proactively providing them because primary care physicians in Korea are not adequately trained to provide these key components of service. Most physicians in Korea are specialists and a great majority of these specialists are engaged in primary care [14]. Yet, throughout their specialist training, they did not learn how to build and maintain relationships with patients in the primary care setting or what services they need to coordinate and provide continuously. This implies that medical training in Korea is producing over-qualified specialists and under-qualified primary care doctors.

For the coordination function of primary care, the main reason for being hesitant to make patient referrals is concern about losing patients. This is confounded in Korea’s highly competitive environment in which there is fierce competition among clinics and between clinics and hospitals. If the patients that are referred to other providers do not return, the referral function will no longer be needed. Therefore, we must carefully examine how to reconstruct the existing referral system. Moreover, it is essential to urgently resolve the problem that the national health insurance system provides little or no economic compensation or incentives for providing coordination, comprehensiveness and continuity of primary care.

This qualitative approach has a limitation in that the findings cannot be generalized to other populations with different personal characteristics, medical treatment experiences, and residential locations. It would be valuable to repeat this study in other settings but due to limited funding and time, we were only able to conduct one FDG for patients and physicians, respectively. Based on the study results, conducting a questionnaire survey might be a good next step to broaden our understanding.

## Conclusion

Lee et al. defines primary care in Korea as follows: “Primary care is the delivery of those health care services that are first encountered by people. It is a discipline in which physicians, who see patients personally in the context of family and community, continue a doctor-patient relationship over time, coordinate health care resources appropriately, and resolve common health care needs of people. To perform the function of primary care effectively, multidisciplinary cooperation and community participation are required [4]”. However, our study showed that the two key players, patients and physicians, do not correctly understand this definition. The evaluation of primary care between these groups also varied.

There are several steps that should be taken to promote primary care in Korea based on these circumstances. First of all, we should undertake efforts to improve the quality of primary care provided by physicians. Some examples are to offer various training activities to improve the quality of care that physicians provide and to develop and utilize clinical practice guidelines. Such training programs should not be exclusive to providing simple medical knowledge but also help primary care physicians accurately understand the functions of primary care, such as coordination, comprehensiveness, continuity; so they can incorporate these functions in their service to patients. Also, the general public should be informed about using clinics instead of hospitals for the treatment of simple or minor diseases. Third, a new compensation scheme should be introduced to compensate physicians for services related to health education, disease prevention, behavioral change and nutrition consultation. Finally, additional reimbursement should be provided to primary care physicians for extending their office hours.
